# Implementation of a New Food Picture Database in the Context of fMRI and Visual Cognitive Food-Choice Task in Healthy Volunteers

**DOI:** 10.3389/fpsyg.2019.02620

**Published:** 2019-11-26

**Authors:** Yentl Gautier, Paul Meurice, Nicolas Coquery, Aymery Constant, Elise Bannier, Yann Serrand, Jean-Christophe Ferré, Romain Moirand, David Val-Laillet

**Affiliations:** ^1^INRA, INSERM, Univ Rennes, CHU Rennes, NuMeCan, Nutrition Metabolisms Cancer, Rennes, France; ^2^CNRS, INRIA, INSERM, IRISA UMR 6074, Empenn – ERL U 1228, University of Rennes, Rennes, France; ^3^Department of Radiology, CHU Rennes, Rennes, France

**Keywords:** eating habits, decision making, brain, internal conflict, healthy subjects

## Abstract

This pilot study aimed at implementing a new food picture database in the context of functional magnetic resonance imaging (fMRI) cognitive food-choice task, with an internal conflict or not, in healthy normal-weight adults. The database contains 170 photographs including starters, main courses, and desserts; it presents a broad-spectrum of energy content and is provided with portion weight and nutritional information. It was tested in 16 participants who evaluated the energy density and gave a liking score for all food pictures *via* numerical scales. First, volunteers were segregated into two groups according to their eating habits according to a food consumption frequency questionnaire (FCFQ) to assess whether the database might elicit different appreciations according to individual eating habits. Second, participants underwent fMRI cognitive food-choice task (van der Laan et al., 2014), using our picture database, in which they had to choose between high-energy (HE) and low-energy (LE) foods, under a similar liking (SL, foods with similar hedonic appraisals) condition or a different liking (DL, foods with different hedonic appraisals) condition. Participants evaluated correctly the caloric content of dishes (from *r* = 0.72 to *r* = 0.79, *P* < 0.001), confirming a good perception of the caloric discrepancies between food pictures. Two subgroups based on FCFQ followed by a principal component analysis (PCA) and a hierarchical ascendant classification (HAC) were defined, that is, Prudent-type (PTc, *N* = 9) versus Western-type (WTc, *N* = 7) consumers, where the WTc group showed higher consumption of HE palatable foods than PTc (*P* < 0.05). The WTc group showed a higher correlation between liking and caloric evaluation of the food pictures as compared to PTc (*r* = 0.77 and *r* = 0.36, respectively, *P* < 0.001), confirming that food pictures elicited variable responses according to contrasted individual eating habits. The fMRI analyses showed that the DL condition elicited the activation of dorsal anterior cingulate cortex (dACC), involved in internal conflict monitoring, whereas SL condition did not, and that LE food choice involved high-level cognitive processes with higher activation of the hippocampus (HPC) and fusiform gyrus compared to HE food choice. Overall, this pilot study validated the use of the food picture database and fMRI-based procedure assessing decision-making processing during a food choice cognitive task with and without internal conflict.

## Introduction

Weight gain is the result of a prolonged energy imbalance, leading to fat mass accumulation. In the most severe cases, the use of bariatric surgery can provide an effective solution to ensure weight loss, but this strategy is not always efficient, involves a risk for significant complications associated with severe dietary constraints in operated patients, and in some instances the emergence of new forms of addiction (e.g., alcohol) (King et al., 2012).

Alternative strategies, such as cognitive-behavioral or brain modulation therapies, aim at regaining control over the emotional and motivational processes. The development of these alternative strategies aim at modifying food choices and decision toward healthy habits and is essentially based on reshaping and reeducating the neurocognitive mechanisms involved in reward processing and food motivation, which are known to be impaired in addict [according to Yale Food Addiction Scale (YFAS) (Gearhardt, 2011)] and obese subjects (Stice et al., 2008; Brooks et al., 2013). Many studies have described the parallel between the abuse of fatty/sweet foods, obesity, and drugs of abuse and their similar impact on the neural circuitry of pleasure, consumption behavior, and health in general (Levine et al., 2003; Volkow et al., 2013).

van der Laan et al. (2014) showed that healthy, that is, normal-weight, weight-concerned women did not experience any conflict in an eliciting self-control dilemma condition (tasty food temptation) contrary to common assumption. These results suggest that failure in self-control could be explained by a lack of internal conflict experience in daily life. Thereby, the self-regulation theory supposes an internal conflict experience when facing a food choice challenging a long-term limited intake goal (Fishbach et al., 2003). Thus, we do not know yet to what extent the chronic consumption of palatable foods (independently of weight gain) can condition the emergence of neuronal plasticity and the implementation of the *shift* (from “controlled eating” to “uncontrolled or habitual eating”) within the hedonic processes and cognitive systems that regulate food choices and intake.

Food picture databases are a widespread tool to assess brain responses to external food visual cues in normal and pathological conditions. Several food picture databases already exist, such as Food-pics from Blechert et al. (2014, 2019), FoodCast research image database (FRIDa) from Foroni et al. (2013), or the image database from Charbonnier et al. (2016). Each food picture database gathers dishes and food items usually encountered in a specific country or culture. Even though the Food-pics or FRIDa databases are valuable tools for research, they do not fit very well with eating habits in foreign countries and mostly present simple food items and not complex dishes. To our knowledge, no existing food picture database has the potential to fit with French eating habits and match with typical dishes and food items encountered in institutional catering. To avoid cultural bias inherent to foreign database and fill this gap, we aimed at providing a new food picture database designed to match with local typical French cuisine in institutional catering and to offer a broad-spectrum of energy content and complex dishes. Many classical dishes should also be familiar to consumers from different Western European countries. The objective of this pilot study was to implement the use of this new food picture database in the context of fMRI and a cognitive food-choice task based on food pictures and the ability of consumers to recognize the caloric density of complex dishes independently from their own eating habits. To test the relevance of pictures for further neurobehavioral studies, (1) real versus estimated (by our volunteers) caloric density was compared to check the good perception of energy variations among food pictures; (2) then, the correlation between liking and energy evaluation of two volunteer groups was performed as control of database relevance according to different eating habits (i.e., high or low consumers of palatable high-calorie foods); and (3) pictures were implemented in fMRI wanting tasks.

The fMRI wanting task was designed to elicit, in half of the situations, an internal conflict when individuals had to choose between a HE and a LE food. Alternation between conflict and conflict-free situations corresponded, respectively, to DL and SL fMRI conditions. Well-segregated brain responses were expected in both SL and DL conditions, and between HE and LE choices to validate this procedure: DL condition was hypothesized to involve conflict monitoring areas, such as the cingulate cortex (Botvinick et al., 1999; Carter et al., 2000; Paus, 2001; Walton et al., 2003), whereas choices without dilemma, that is, SL condition, may activate more the working memory areas, such as the dorsolateral prefrontal cortex (dlPFC) (Fletcher, 2001). Even though dyadic picture choice task is known to be more complex than single-picture evaluation and subjected to potential biases related to visual preferences or image structure discrepancies (Krajbich et al., 2010; Sokol-Hessner et al., 2012), this paradigm is frequently used to assess food preferences and decisions in human volunteers and patients. Moreover, the dyadic picture choice paradigm is a way to investigate internal conflict (the subjects have to deal with two incompatible drives during the food choice, that is, high palatable food preference against LE healthy food), which is one the ultimate goals of our research.

## Materials and Methods

### Ethics Statement

This study was approved by the Ethics Committee Ouest V, Rennes (*Comité de Protection des Personnes Ouest V*), reference number: 11/43-832, study number: 2011-A01531-40. Prior to experiment, subjects were given detailed information about the procedure and provided written informed consent. Participants were unpaid for their participation. Only travel expenses were refunded in the form of bus tickets.

### Food Picture Database Building

#### Food Pictures Acquisition

Food pictures were created and selected to constitute a food picture database typical of food choices offered in French institutional collective catering. Food pictures were taken every day during a 4-month period (September–December) in a well-known French collective catering chain (Restauration Collective Casino, R2C). A total of 170 courses were included in the database, representing the diversity of dishes encountered during the collection period. Pictures of starters, main courses, and desserts were taken with a standardized setup. A Nikon D800 DSLR with AF-S 50 mm f1.4 fixed focal lens was mounted on a tripod oriented 45° down. Dishes were put in a 50 cm × 50 cm × 50 cm white shooting chamber with dark blue background. Two Nikon SB600 flashes were positioned at 20 cm on each side of the chamber. Pictures were taken in manual mode, with a 1/250s exposure time, and f10 aperture at ISO 100 sensitivity. The setup ensured both enough depth of field and negligible ambient light compared to flash light. The photographs were saved in NEF raw format. RawTherapee 4.2 (Horváth, 2014) software was used to crop and convert pictures in png format in standard sRGB color space. White balance was set around 5400K using spot gray level settings. Pixelmator 3.4 (Dailide and Dailide, 2015) was used to replace the background of all images by a uniform gray value of 125.

#### Real Energy Determination

Each course was weighted for caloric evaluation. When required, the main protein course (e.g., meat, fish, omelet, etc.) and side dish (e.g., vegetables, starchy foods, etc.) were weighted separately. Caloric density was evaluated using the Ciqual 2013 database (The French Information Center on Food Quality, 2013) and expressed in kilocalories. This database provides nutritional information on traditional French food items and courses. For a few courses with no equivalent in Ciqual 2013 database, USDA National Nutrient Database for Standard Reference (United States Department of Agriculture Agricultural Research Service [USDA], 2015) was used.

#### Image Metadata

The metadata related to the 170 food pictures are available in the Supplementary Table S1. Among the different parameters provided are the type of dishes, their names in French and English, net weight, and total calories, as well as image characteristics including color balance, object size, intensity, and complexity. The scripts provided by Blechert et al. (2014) were used to generate these images parameters.

### Participants

Participants were 16 healthy men and women living in France for at least 1 year (sex ratio = 50/50), aged 29.2 ± 6.2 years, with an average BMI of 21.3 ± 2.3. The participants met the following inclusion criteria: between 18 and 40 years old, normal BMI between 18 and 25, right-handed. All of the participants were Caucasian and native French speakers. Half of the participants were recruited in our research institute among students and scientists. Others had heard about the study and had contacted the research group for participation. Only six participants over 16 had a background in nutrition.

#### Recruitment

Participants were excluded if athletes, persons with substance use or eating disorders (tobacco, alcohol, drug, food–bulimia/anorexia), food taboos–whether for ideological (vegetarianism, veganism), religious or health (allergy, intolerances) reasons–pregnant women, people without health insurance, and/or insufficient knowledge of the French language. These selection criteria were chosen to recruit a relatively homogeneous group of healthy volunteers without any addiction or eating disorder and who could understand clearly the instructions and evaluate the food pictures without any extreme beliefs or taboos related to food consumption.

Participants were selected using the following self-administered paper questionnaires. The Ricci & Gagnon questionnaire assessed physical activity levels, and participants were excluded in case of high physical activity level representing by a score > 32 (Ricci and Gagnon, 2011). The AUDIT questionnaire assessed drinking patterns and excluded excessive drinkers defined as score >7 for women and >8 for men (Saunders et al., 1993). The CRAFFT questionnaire assessed substance-related risks and problems in adolescents, excluding participants if score >2 “yes” (Knight et al., 2002). The SCOFF questionnaire assessed the possible presence of an eating disorder; participants were excluded if score >1 “yes” (Morgan et al., 1999). Questions related to smoking and e-cigarette consumption excluded daily smokers because of nicotine impact on eating behavior and sensory abilities; occasional smokers were included (consumption frequency < 1/day).

#### Eating Habit-Based Group Definition

The 16 selected participants were asked to complete a FCFQ (Deschamps et al., 2009). The questionnaire was computerized under TypeForm^®^ online free software (Muñoz and Okuniev, 2012). In our study, we chose to conserve only the palatable food items (*N* = 56, no picture, text only) that are usually associated with risky eating habits and of which the consumption frequency must remain low according to the French PNNS (Program National Nutrition Santé–National Nutrition & Health Program). The rationale was to discriminate our volunteers according to their consumption frequency of these specific food items. As a consequence, the “unhealthy” palatable food items included high-fat high-sugar/salt processed foods (e.g., burgers, pizzas, delicatessens, cheeses, and candies, etc.) but also meat because high consumption of (red and processed) meat is associated with many diseases, such as cancer, overweight, and metabolic disorders (Micha et al., 2010). Natural and non-processed food items (e.g., fruits, vegetable, and unprocessed cereals, etc.) were excluded from the restricted FCFQ used in this study.

This questionnaire assessed food eating frequency during the last 12 months, asking for each food item “*In the past 12 months, how often did you consume*…*?*”

The frequency responses were defined and scored as follows: never (scored 0), fewer than once per month (scored 0.5), 1–3 times per month (scored 2), once per week (scored 4), 2–5 times per week (scored 14), once per day (scored 30), and several times per day (scored 60), corresponding to a monthly consumption frequency.

The 56 food items were pooled within seven categories: Offal/Cold meat/Eggs, Meat, Processed foods, Cheese, Dairy products, Candies/Appetizers, Ice cream and Fat (see Table 1 for details). Items’ scores were averaged to obtain a mean score for each of the seven food categories for each volunteer. The obtained matrix was analyzed by PCA followed by a HAC with an optimal number of classes adjusted between two and six and clustering consolidation to discriminate a Western-type (WTc) and a Prudent-type consumer (PTc) group, respectively, with high- versus low-consumption frequency of palatable foods. Then, Wilcoxon-Mann-Whitney tests were performed to compare eating frequencies between groups for each of the seven food categories.

**TABLE 1:** Food categories composition.

**Food category (*N* = 7)**	**Items (*N* = 56)**
Offal, cold meat, eggs	Offal, ham, saucisson, cervelas/mortadelle, pate/rillette, sausage, hard-boiled eggs, fried eggs
Meat	Beef, chopped steak, pork, veal, poultry, ovine
Processed food	Roll parcel, quiche, Croque-Monsieur, pizza, raviollis, hamburger, french fries, cassoulet, couscous, sauerkraut, paëlla, chili con carne
Cheese, dairy product	Emmental, grated cheese, babybel/gouda/edam, brie/camembert, bleu cheese/roquefort, yogurt, dessert
Candy and appetizers	Honey, marmalade, sugar, cocoa powder, chocolate, pastries, pies, brioche, candy, biscuits, cake, cereal bars, peanuts, crisps
Ice cream	Ice cream, chantilly, sorbet
Fat	Butter, margarine, mayonnaise, vinaigrette, ketchup, fresh cream

#### Energy Evaluation

The 16 participants were asked to estimate the caloric content of all food pictures, answering the question “*In your opinion, the starter/main course/dessert shown in this photo is*…,*”* using a 10-point numerical scale, ranging from 1: “*Not at all caloric*” to 10: “*Very caloric.*” The questionnaire was computerized under TypeForm^®^ online free software (Muñoz and Okuniev, 2012).

#### Analysis

Spearman’s correlation tests were performed to investigate the relationship between real and subjectively assessed (evaluated) energy content.

#### Liking (i.e., Hedonic) Evaluation of the Food Pictures

Participants completed a hedonic evaluation answering the question “*Indicate how much you like this starter/main course/dessert using the numeric scale from 1 to 10 at the bottom of the screen*,” ranging from 1: “*I really don’t like it*” to 10: “*I really like it*,” for each of the 160 food pictures from our database.

#### Analysis

Spearman’s correlation tests were performed to investigate the relationship between subjectively assessed (evaluated) energy content and liking scores in both WTc and PTc groups.

### fMRI Wanting Task

The grades from the liking evaluation were used to determine individual pairing of food pictures for the fMRI wanting task to create two conditions: an SL condition and a DL condition. Each two-choice task in the fMRI was consequently individually designed on the basis of individual hedonic ratings. The liking questionnaires used in this study were computerized under TypeForm^®^ online free software (Muñoz and Okuniev, 2012).

#### Liking Task Analysis

Spearman’s correlation tests were performed to investigate the relationship between hedonic and previous evaluated energy content.

#### Building of Similar and Different Liking Conditions

**FIGURE 1:**
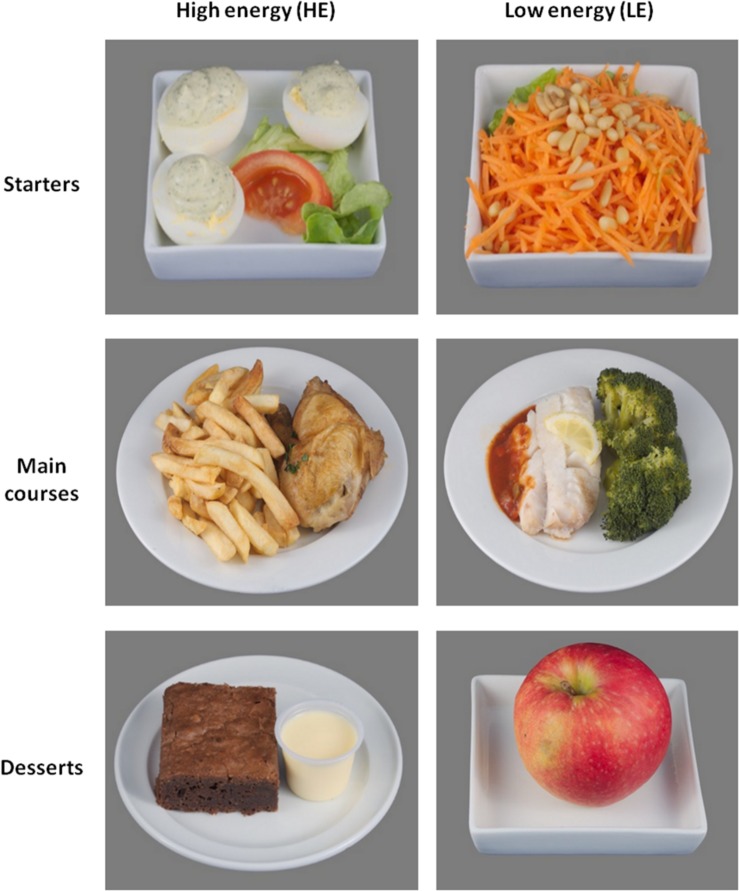
Example of food pictures pairs, extracted from our food picture database, according to categories (starters, main courses, and desserts) and energy content [high-energy (HE) food choice and low-energy (LE) food choice].

The SL condition proposed pairs of pictures having received either exactly the same grade or presented no more than one point of gap according to the hedonic evaluation. For example, for the SL condition, in one pair of pictures, picture 1 was graded 8 versus picture 2 graded 8 or 9 or 7. The DL condition proposed pairs of pictures having received grades presenting two to three points of gap. For example, for the DL condition, in one pair of pictures, picture 1 was graded 8 versus picture 2 graded 6 or 5 or 10. In the SL condition, as in the DL condition, there was always a factor-2 caloric gap between associated pictures determining an HE versus an LE food choice (example in Figure 1). In the DL condition, the food item with the highest liking grade (i.e., the preferred food) was always the HE food item to create a cognitive conflict between the food items’ healthiness and palatability. Pictures pairing was automatized for each participant, thanks to an Excel macro applied on individual hedonic evaluation results.

#### Wanting Task Procedure

**FIGURE 2:**
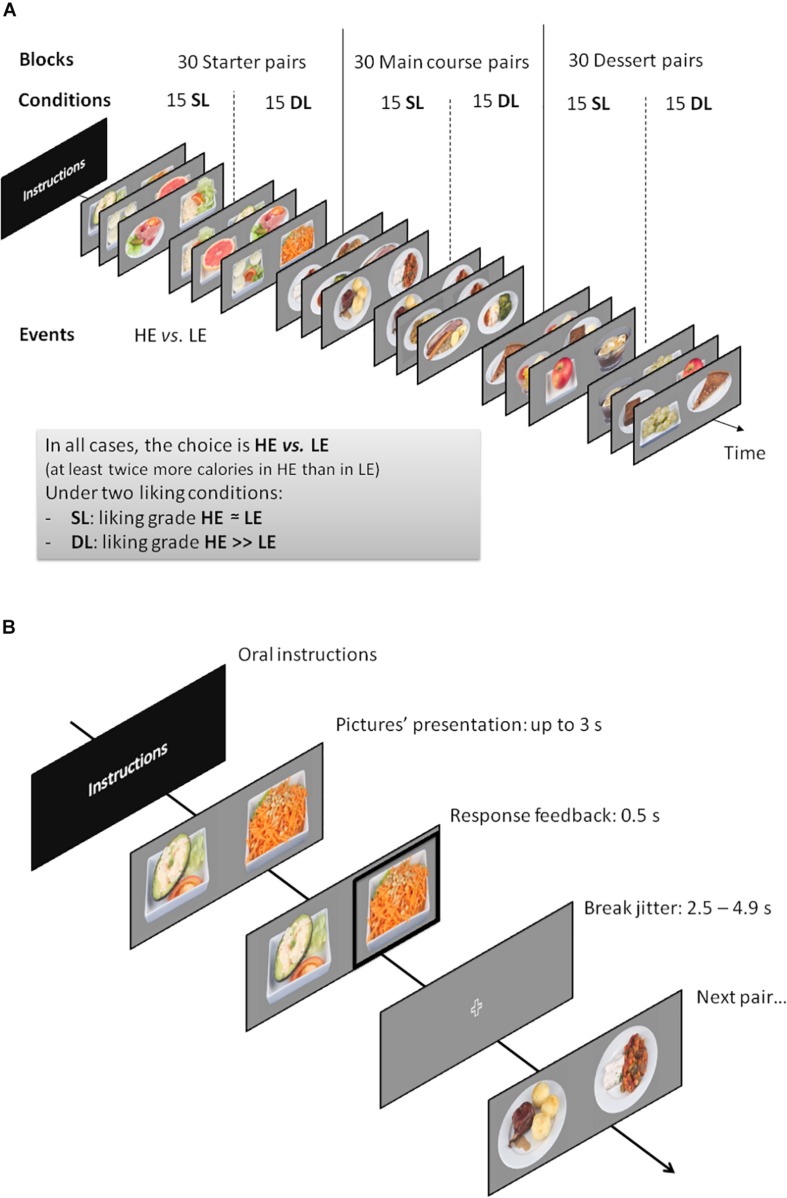
**(A)** Functional MRI (fMRI) task general work plan. SL, similar liking condition; DL, different liking condition; HE, high-energy food choice; LE, low-energy food choice. **(B)** The fMRI paradigm adapted from van der Laan et al. (2014).

The general fMRI procedure was the same as that described by van der Laan et al. (2014) and consisted of one-session event-related task including three successive blocks (one per category: starter, main course, desserts) with two conditions (SL and DL) and choice events (HE and LE). Ninety pairs of food pictures from our database, all different and equally divided between starters, main courses, and desserts, were successively shown on a screen. For each pair, subjects had to choose between an HE versus an LE food, the food item they would prefer to eat for lunch on the same day. The side on which the LE or the HE food items were shown in pairs was randomized. For each category (starter, main course, and desert), the first 15 pairs of pictures corresponded to the SL condition, and the 15 further pairs corresponded to the DL condition without participants’ awareness (Figure 2A). Each pair of pictures was showed for up to 3 s using an event-related design. A visual feedback (bold frame contouring the chosen food item) was displayed during 0.5 s after the subjects’ answers (using a response grip in their right hand). If no answer was given during the allocated 3-s time, a message “*too late*” was displayed instead of the feedback. Then, a white cross on gray background was shown as a neutral screen during a jitter time comprised between 2,500 and 4,900 ms (Figure 2B). The jitter between two successive pairs of pictures (i.e., the rest time with neutral screen between two successive choices) was randomized between choices, and it was identical for all participants.

#### Wanting Task Analysis

Choice frequencies were analyzed using a paired *t*-test to investigate energy content effect (HE vs. LE) within each condition (SLLE vs. SLHE and DLLE vs. DLHE) and condition effect (SL vs. DL) on choice type (SLLE vs. DLLE and SLHE vs. DLHE). All acronyms for comparisons are described in Table 2. Response delay was analyzed using a paired *t*-test to investigate condition effect (SL vs. DL) and energy-type choice effect (HE vs. LE). All tests have been done for starters, main courses, and desserts pooled together and separately.

**TABLE 2:** Acronyms used in statistical comparisons.

**Acronyms**	**Definition**
SL	Similar liking condition
DL	Different liking condition
HE	High-energy food choice
LE	Low-energy food choice
SLHE	High-energy food choice made in SL condition
SLLE	Low-energy food choice made in SL condition
DLHE	High-energy food choice made in DL condition
DLLE	Low-energy food choice made in DL condition
Acronyme_Starters/Main/Dessert_	Energy/condition criteria for starters, main courses, or desserts food category
WTc	Western-type consumer group
PTc	Prudent-type consumer group

### Brain Activation Analysis

#### Preparation of the Subjects

The participants had to fast for at least 3 h before fMRI, which was performed between 11:00 am and 12:30 pm (before lunch). In the MRI scanner, the participants were positioned head-first supine. A mirror was fixed on the head coil above the participants at eye level to allow the visualization of a screen at the rear of the scanner, on which the task was presented. The participants were able to make choices with a response grip placed in their right hand, lying on their right thigh, so that the thumb was on the left and the index was on the right. Before the task, participants received orally the corresponding task instructions.

#### fMRI Acquisition

Acquisitions were performed on a 3T Siemens MRI (SIEMENS Magnetom Verio syngo MR B17) using a 32-channel phased array coil. The Nordic Neurolab solution (Bergen, Norway) and EPrime 2.0.8 Professional (PST, Sharpsburg, MD, United States) were used to display the visual task *via* a rear-facing mirror placed on the head coil. An anatomical sequence (T1 3D MP-RAGE, FOV = 256 mm^2^ × 256 mm^2^, 176 slabs, TR = 1,900 ms, TE = 2.26 ms, TI = 900 ms, flip angle = 9°, voxels size = 1 mm^3^ × 1 mm^3^ × 1 mm^3^) and an axial T2 FLAIR sequence were acquired to detect a potential brain abnormality, in which case the subject was excluded from the analysis. fMRI with a sensitive T2-weighted gradient echoplanar imaging sequence with 36 slices of 3 mm was acquired during the 12-min cognitive task. The acquisition parameters were FOV = 192 mm^2^ × 192 mm^2^, EPI factor 64, voxels size = 3 mm^3^ × 3 mm^3^ × 3 mm^3^, TR = 2,000 ms, TE = 30 ms, and flip angle = 90°.

#### fMRI Analysis

Images analysis was performed using the Statistical Parametric Toolbox 8 (Wellcome Department of Imaging Neuroscience, London, United Kingdom) in Matlab (Mathworks, Inc., Sherborn, MA, United States). Functional images were realigned to the mean to correct for motion during the acquisition and coregistered on 3D T1 anatomical images. The anatomical and functional images were then normalized to the Montreal Neurological Institute (MNI, McGill University, Montreal, QC, Canada) space. Spatial smoothing of functional images was performed with a 6-mm full width at half maximum (FWHM). One subject was removed from fMRI analysis because of movements exceeding the size of one voxel. Vector onsets were compiled for the SL, DL, HE, and LE conditions described above. A general linear model analysis was performed using canonical hemodynamic response function (HRF) with time and dispersion derivatives as proposed by SPM8 (Statistical Parametric Mapping, Wellcome Trust Centre for Neuroimaging, University College London, London, United Kingdom). Two separate analyses were performed, the first with SL and DL conditions (Model 1), the second with LE and HE subject choices (Model 2), both considering direct delta functions. Contrasts analyzed were SL versus DL to test condition effect and investigate potential internal conflict in DL condition, and HE versus LE to test the effect of energy content on brain processes. These two comparisons represented two distinct models. We also investigated independently brain responses to SL and DL in the first model and to HE and LE condition/choice in the second model. All of these tests were performed using whole-brain approach. Statistical significance was assessed for cluster-wise significance defined by random field theory (corrected *p* = 0.05) to take into account the spatial autocorrelation. Because of the small size of the cohort, WTc versus PTc (*N* = 9 and *N* = 7, respectively) group comparison was not performed for fMRI data in this study.

Statistical analyses were performed with the R 3.1 software (University of Aalborg, Denmark).

## Results

When required, results are presented as mean ± SEM.

### Food Picture Database

#### Description

**FIGURE 3:**
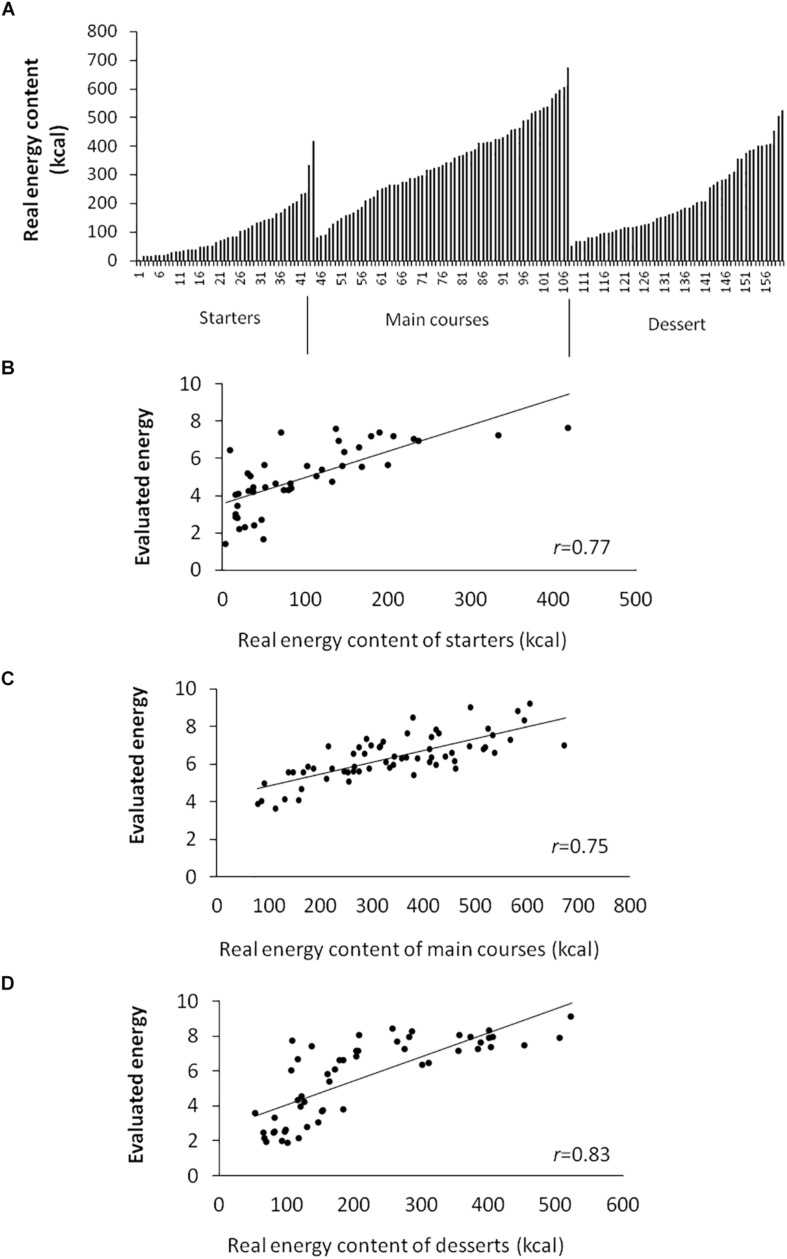
**(A)** Energy content repartition (in kilocalories) among starters, main courses, and desserts among the 164 food pictures from the database. **(B)** Evaluated energy content (scored between 1 and 10) according to real energy content (in kilocalories) of starters (Spearman’s correlation *r* = 0.77, *P* < 0.001), **(C)** main courses (Spearman’s correlation *r* = 0.75, *P* < 0.001), and **(D)** desserts (Spearman’s correlation *r* = 0.83, *P* < 0.001).

The food picture database provides 170 food pictures, including 44 starters, 63 main courses, and 57 desserts with nutritional information (extract in Table 3), as well as 6 additional pictures with cheeses. The database provides a broad spectrum of energy content (from 4 to 673 kcal for the entire database) in each food category (Table 4 and Figure 3A).

**TABLE 3:** Extract from nutritional information of the food picture database (the whole database if composed of 170 food items).

**Category**	**Dish name**	**Net weight (g)**	**Kcal/100 g**	**Total calorie (kcal)**
Starter	Country ham	44	232	102
	Lentils	116	114	132
	Celery stick and celeriac	70	26	18
Main course	Cod loin with tomato sauce and broccoli	239	66	159
	Cheese tartiflette	421	144	606
	Burger au gratin and rice	175	196	343
	Chicken and French fries	276	211	583
Dessert	Crème caramel	143	136	194
	Grape	140	70	98
	Chocolate mousse	66	164	108
	Creamy yogurt	125	122	153
	Banana	124	94	116
	Floating island	99	138	137

**TABLE 4:** Food pictures database characteristics.

	***N***	**Net weight (g) (min–max)**	**Kcal/100 g (min–max)**	**Total kcal (min–max)**	**Spearman’s correlation between real (kcal) and evaluated energy content**	
					
					***r***	***p-value***
Starters	44	25–450	14–402	4–418	0.77	<0.0001
Main courses	63	460–422	42–233	79–673	0.75	<0.0001
Desserts	57	51–235	42–504	53–523	0.83	<0.0001
Total	164	25–422	14–504	4–673	0.75	<0.0001

#### Energy Content Evaluation

Results from all participants pooled together (*N* = 16) showed a strong correlation between real and evaluated energy content for starters, main courses, and desserts combined (*r* = 0.75, *P* < 0.0001) and independently (*P* < 0.0001 for all, Figures 3B–D).

### Eating Habit-Based Group Definition of Participants

**FIGURE 4:**
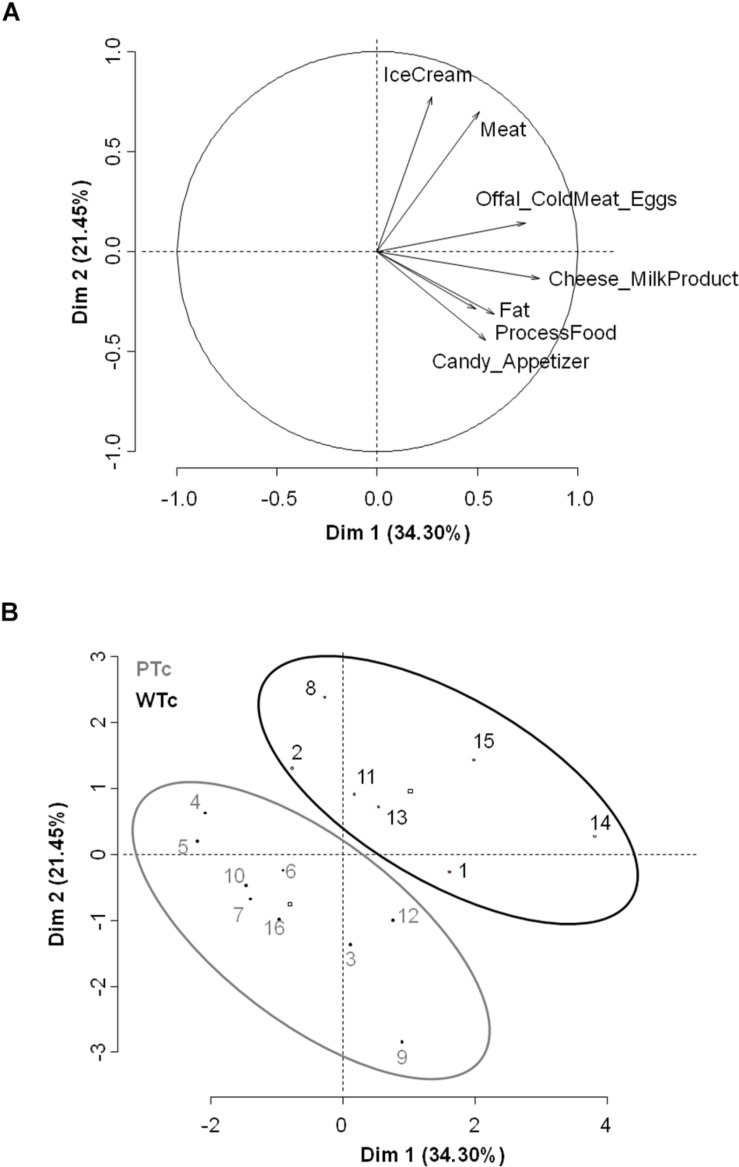
**(A)** Variables factor map from principal component analysis (PCA) on Food Frequency Consumption Questionnaire. **(B)** Individuals’ graph from hierarchical ascendant classification (HAC) analysis discriminating both Western-type consumer (WTc, *N* = 7, black) and Prudent-type consumer (PTc, *N* = 9, gray) groups (number of clusters required: 2–6, with clustering consolidation).

The PCA analysis performed on FCFQ showed that Offal/Cold meat/Eggs associated with Cheese consumption contributed to axis 1 for 22.7% and 27.3%, respectively. The axis 2 was built from contribution of Meat and Ice cream consumption for 32.5% and 39.9%, respectively (Figure 4A).

Two groups were built according to HAC: PTc (*N* = 9, five women and four men) and WTc (*N* = 7, three women and four men) (Figure 4B). Groups were similar in terms of age (PTc: 27.2 ± 1.5 years, WTc: 31 ± 2.8 years, *F*_1_,_14_ = 1.56, *P* = 0.23), and BMI (PTc: 21.9 ± 0.8 kg/m^2^, WTc: 21.3 ± 0.8 kg/m^2^, *F*_1_,_14_ = 0.38, *P* = 0.55).

**FIGURE 5:**
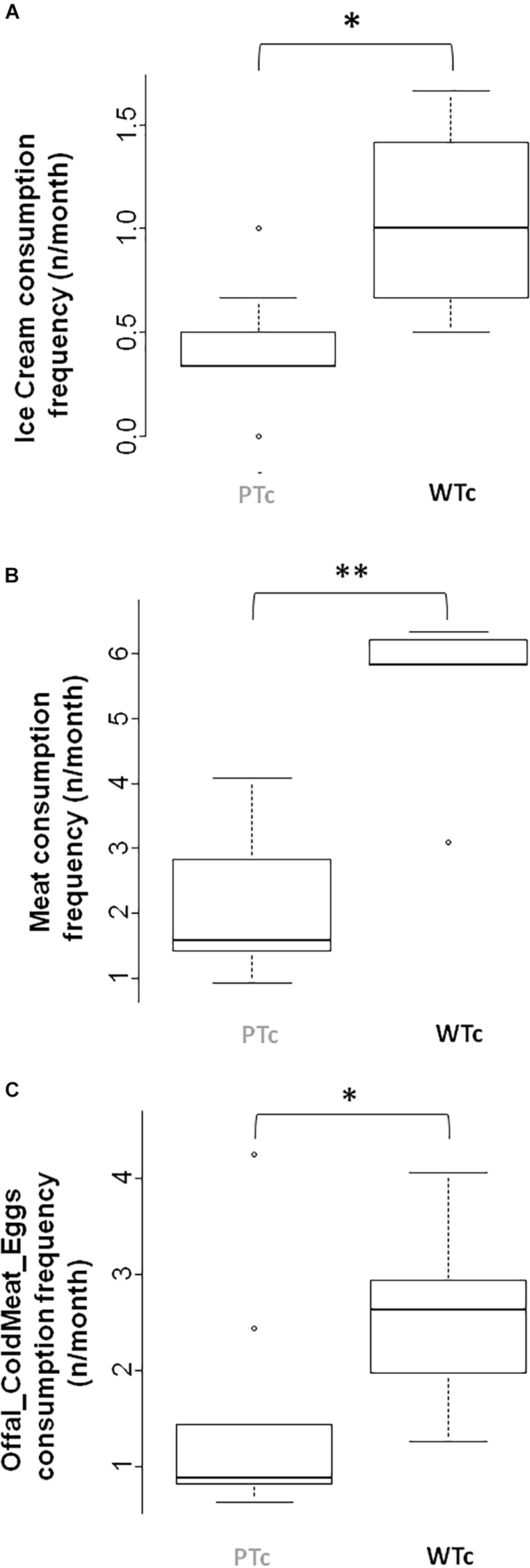
Palatable food frequency consumption in both Prudent-type consumer (PTc) and Western-type consumer (WTc) groups for **(A)** Ice cream (*W* = 13, ^∗^*P* < 0.05), **(B)** Meat (*W* = 4, ^∗∗^*P* < 0.01), and **(C)** Offal, Cold meat, and Eggs (*W* = 8, ^∗^*P* < 0.05) food category.

Statistical Wilcoxon test analysis showed that the WTc had a higher eating frequency of Ice cream (*W* = 13, *P* < 0.05; Figure 5A), Meat (*W* = 4, *P* < 0.01; Figure 5B), and Offal/Cold meat/Eggs (*W* = 8, *P* < 0.05; Figure 5C) than the PTc. No difference in other food-eating frequency was found between the groups.

### Data Base Evaluation

**FIGURE 6:**
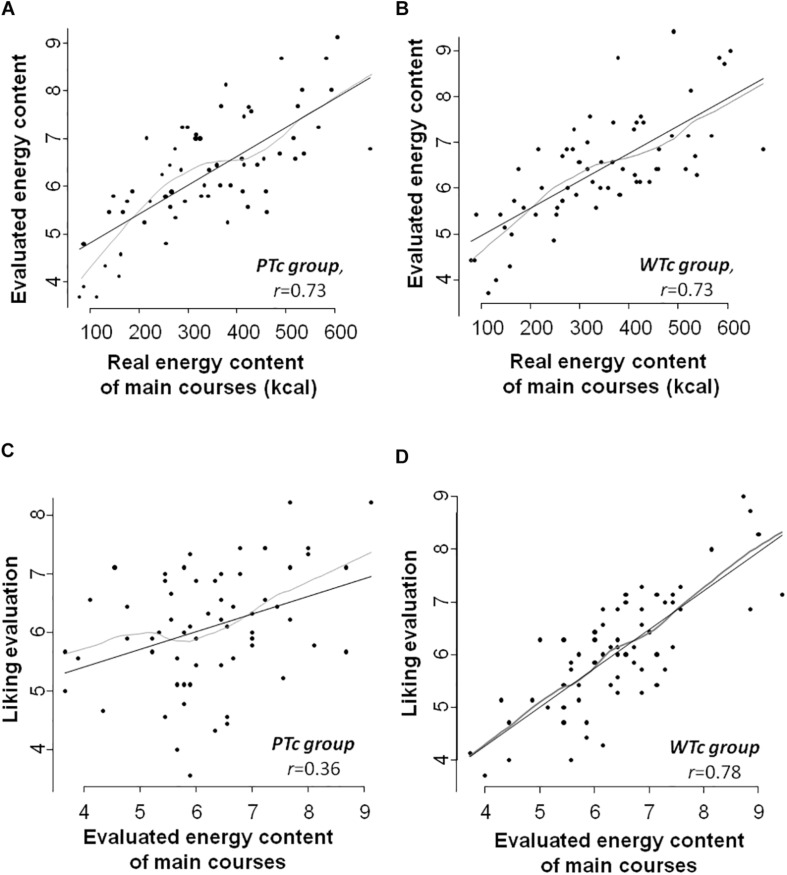
Top: Evaluated energy (scored between 1 and 10) according to real energy content of main courses (in kilocalories) pictures presented to participants in both **(A)** prudent-type consumers (PTc) group (Spearman’s correlation *r* = 0.73, *P* < 0.001) and **(B)** western-type consumers (WTc) group (Spearman’s correlation *r* = 0.73, *P* < 0.001). Bottom: **(C)** Liking evaluation (scored between 1 and 10) according to evaluated energy of main courses pictures (scored between 1 and 10) in PTc (Spearman’s correlation *r* = 0.36, *P* < 0.01) and **(D)** WTc (Spearman’s correlation *r* = 0.78, *P* < 0.001) groups.

Strong correlations between real and evaluated caloric content were observed in both WTc and PTc groups for starters (*r* = 0.72, *P* < 0.001 and *r* = 0.73, *P* < 0.001 for PTc and WTc, respectively), main courses [*r* = 0.73, *P* < 0.001 and *r* = 0.73, *P* < 0.001 for PTc (Figure 6A) and WTc (Figure 6B), respectively], and desserts (*r* = 0.75, *P* < 0.001 and *r* = 0.80, *P* < 0.001 for PTc and WTc, respectively).

#### Correlation Between Evaluated Energy Content and Liking

Correlations between evaluated caloric content and liking were poor or non-significant, even for starters and desserts: the PTc showed poor to moderate negative correlation between liking and energy content (*r* = −0.46 and *r* = −0.39, *P* < 0.01 for starters and desserts, respectively), and the WTc showed no correlation between these items (*r* = −0.03, *P* = 0.85 and *r* = 0.23, *P* = 0.10 for starters and desserts, respectively). Concerning the main courses, PTc showed a poor correlation between liking and caloric estimation with *r* = 0.36 (*P* < 0.01; Figure 6C), whereas WTc showed a strong correlation between these variables (*r* = 0.78, *P* < 0.001; Figure 6D).

### fMRI Wanting Task

#### Choices in fMRI Wanting Task

##### Condition effect

Compared to the SL condition, the DL condition led to a higher frequency of HE main courses choices (DLHE: 9.8 ± 0.7 vs. SLHE: 8.3 ± 0.4, *t* = 2.67, df = 15, *P* < 0.05).

##### Energy content effect

In the DL condition, HE main courses were chosen more frequently than LE ones (DLHE: 9.7 ± 0.7 vs. DLLE: 5.2 ± 0.7, *t* = 3.13, df = 15, *P* < 0.01), whereas only a trend appeared in the SL condition between HE and LE choice frequencies (SLHE: 8.3 ± 0.4 vs. SLLE: 6.6 ± 05, *t* = 1.81, df = 15, *P* = 0.09).

#### Response Delay in fMRI Wanting Task

##### Conditions comparison

Response delays were longer in the SL compared to the DL condition (SL: 1,562.3 ± 54.4 ms vs. DL: 1,390.4 ± 47.7 ms, *t* = −5.8, df = 15, *P* < 0.001). This was true for starter choices (SL_starters_ = 1,583.6 ± 82.9 ms vs. DL_starters_ = 1,304.2 ± 44.4 ms, *t* = −3.7, df = 15, *P* < 0.001) and dessert choices (SL_desserts_ = 1,495.9 ± 57.9 ms vs. DL_desserts_ = 1,341 ± 57.8 ms, *t* = −3.71, df = 15, *P* < 0.001) but not for main course choices (SL_main_ = 1,607.9 ± 67.8 ms vs. DL_main_ = 1,525 ± 68.6 ms, *t* = −1.6, df = 15, *P* = 0.13).

##### Energy content comparison

No difference appeared in response delays between general HE versus LE choices (HE = 1,477.2 ± 50.6 ms vs. LE = 1,494 ± 53.5 ms, *t* = −0.5, df = 15, *P* = 0.66), but choosing LE main courses took more time than choosing an HE food (HE = 1,519.5 ± 60.9 ms vs. LE = 1,675.7 ± 73.3 ms, *t* = −3.02, df = 15, *P* < 0.01).

### Brain Activation Analysis

**FIGURE 7:**
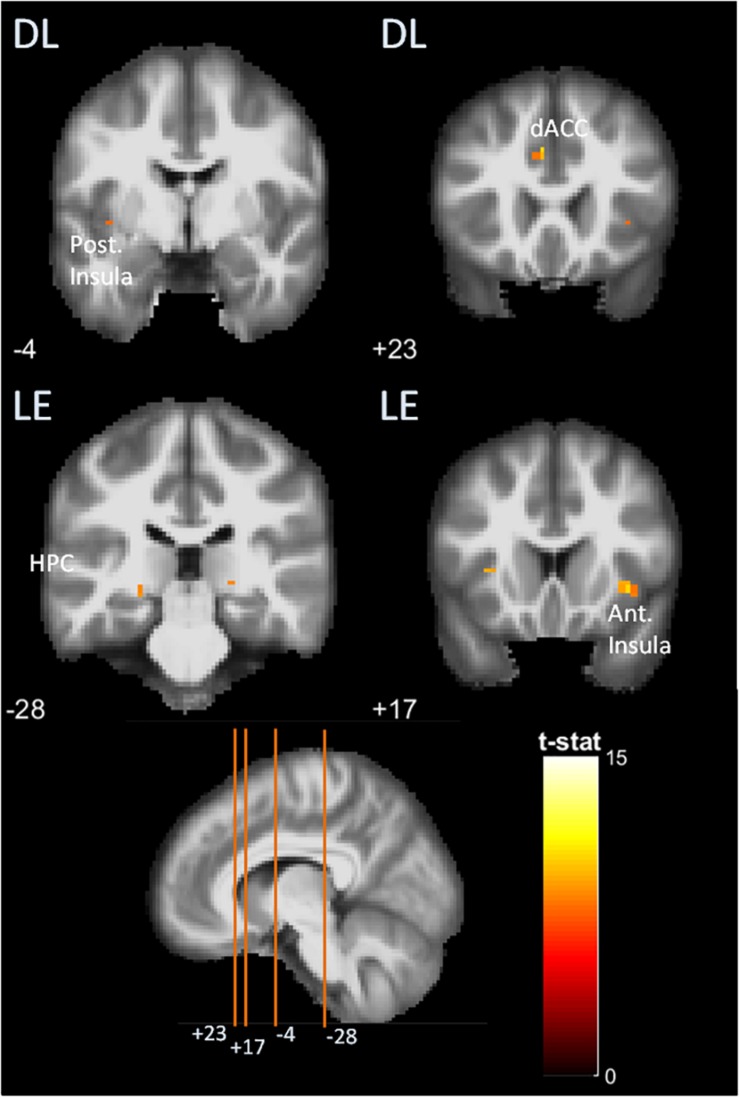
Brain activations detected in functional MRI (fMRI) two-choice task viewed in coronal slices, in the posterior and anterior insula (Post. and Ant. Insula), dorsal anterior cingulate cortex (dACC), hippocampus (HPC), fusiform gyrus (FG), occipital-fusiform complex (LOC), in similar liking (SL) and different liking (DL) conditions and for high-energy (HE) and low-energy (LE) food choices. The bottom left panel indicates the location of the coronal slices on a sagittal slice (+9). MNI coordinates are indicated for each coronal slice.

Brain activities in SL, DL, HE, and LE are available in Table 5 and Figure 7.

**TABLE 5:** First-level analysis. Brain responses to SL and DL conditions and to HE and LE choices in the entire population (*N* = 15).

**Anatomical area**				**MNI Coordinates**			***P*-value**		
		
**Structure**	**Hemisphere**	**BA**	**K**	***x***	***y***	***z***	**FWE**	**FDR**	**Uncorr**
**SL condition (SL = *1; DL* = *0)***									
Secondary visual cortex	L	18	21	−15	−100	−5	<0.001	<0.001	<0.001
Premotor cortex	L	6	2	−27	−7	55	0.002	0.035	0.014
Fusiform gyrus	L	37	16	−30	−55	−20	<0.001	<0.001	<0.001
**DL condition (DL = 1; SL = 0)**									
Anterior insula	R	13	26	36	17	−2	<0.001	<0.001	<0.001
Posterior insula	L	13	2	−39	−4	−2	0.002	0.016	0.010
Secondary Visual cortex	L	18	13	−9	−103	−5	<0.001	<0.001	<0.001
Occipital fusiform gyrus	R	-	17	27	−73	11	<0.001	<0.001	<0.001
Associative visual cortex	R	19	25	24	−52	−8	<0.001	<0.001	<0.001
Premotor cortex	L	6	5	−48	−1	28	<0.001	<0.001	<0.001
Dorsal anterior cingulate	L	32	6	−6	23	34	<0.001	<0.001	<0.001
Visual associative cortex	R	19	2	18	−67	−8	0.002	0.016	0.010
Thalamus	R	50	3	21	−31	1	<0.001	0.006	0.002
**HE condition (HE = 1; LE = 0)**									
Secondary visual cortex	L	18	13	−9	−103	−5	<0.001	<0.001	<0.001
Anterior insula	R	13	5	36	17	−5	<0.001	0.002	<0.001
Occipital fusiform gyrus	R	-	4	27	−73	−11	<0.001	0.004	0.001
Premotor cortex	L	6	2	−45	5	31	0.002	0.024	0.013
**LE condition (LE = 1; HE = 0)**									
Secondary visual cortex	L	18	18	−15	−100	−5	<0.001	<0.001	<0.001
Anterior insula	R	13	10	36	17	−5	<0.001	<0.001	<0.001
	L	13	8	−30	14	4	<0.001	<0.001	<0.001
Visual associative cortex	R	19	27	24	−52	−8	<0.001	<0.001	<0.001
	L	19	6	−21	−64	−8	<0.001	<0.001	<0.001
Occipital fusiform gyrus	R	-	8	27	−73	−11	<0.001	<0.001	<0.001
Supramarginal gyrus	L	40	6	−45	−31	46	<0.001	<0.001	<0.001
Hippocampus	L	54	2	−24	−28	−8	0.002	0.017	0.012
Fusiform gyrus	L	37	17	−30	−55	−17	<0.001	<0.001	<0.001
Thalamus	R	50	3	21	−28	−2	<0.001	0.005	0.003

*Height threshold *T* = 7.76, *P* < 0.05 FWE, df = 1,14, Extent threshold: *k* = 2 voxels, voxels size: 3.0 mm × 0 3.0 mm. L, left; R, right; BA, Brodmann area; K, cluster size (number of voxels); SL, similar liking; DL, different liking; HE, high-energy food; LE, low-energy food; FWE, family-wise error correction; FDR, false discovery rate correction; Uncorr, uncorrected *p*-value.*

### Model 1

#### Response to SL Condition

Brain activation was found in BA18 and BA37 (visual areas) as well as BA6 (premotor cortex).

#### Response to DL Condition

Brain activation was found in the visual associative area and FG (visual areas), in the anterior and posterior insula (gustatory areas), in the cingulate cortex (conflict monitoring area), and thalamus (sensory relay, regulation of consciousness and alertness).

### Model 2

#### Response to HE Choice

Activations were found in the insula (taste integration area) and in the fusiform occipital cortex (visual/attentional areas).

#### Response to LE Choice

Activations were observed in the fusiform occipital cortex (visual/attentional areas), HPC (memory area), supramarginal gyrus (cognitive and judgment area), anterior insula (taste integration area), and thalamus (sensory relay, regulation of consciousness and alertness).

## Discussion

In this study, we built a new freely available food picture database typical of French collective catering and matching with local dietary habits, which is necessary to investigate eating preferences and choices with the possibility to present complex dishes in the context of a complete menu (i.e., starter, main dish, and dessert). Despite the low number of participants included in this pilot study, the analysis of FCFQ allowed us to identify two relevant groups with contrasted eating habits in terms of palatable HE food consumption frequency, that is, PTc (*N* = 9) and WTc (*N* = 7) groups. The most discriminating food categories between groups were the ice cream, offal/cold meat/eggs and meat. Both WTc and PTc correctly evaluated the energy content of the food items, showing a good perception of the widespread caloric content available in the database. Correlation between liking and main courses energy content was strong in the WTc (*r* = 0.78) and poor in the PTc (*r* = 0.36). This result led to the proof of concept that our picture database, containing a wide variety of dishes with high or LE content and various combinations of ingredients/items, elicited the expected different responses among people with contrasted eating habits.

Interestingly, during the fMRI task, we found that the response delay was longer in the SL compared to DL condition, and that LE main courses choice required more time than HE food. We showed that the type of food choices and conditions elicited significantly selective brain responses when all participants were considered (*N* = 15): DL condition elicited dACC and insula activation, whereas SL condition did not. LE food choice activated stronger cognitive processing areas, with occipital FG, HPC, and supramarginal gyrus, compared to HE choices.

This pilot study must be interpreted in the light of its limitations and strengths. The actual food picture database includes mainly autumn-winter dishes and would need summer-type dishes, including seasonal fruits and vegetables, to account for seasonal variations. This will be done in a further augmented version of our database. Also, various diet-specific or cultural-specific dishes should be added to this database in the future to take into account cultural, ethics, or religious motives in food-intake decisions. The FCFQ general approach in nutritional epidemiology was largely used to characterize dietary patterns because of its high reproducibility and validity (Khani et al., 2004), and the questionnaire used in the present study was previously validated (Deschamps et al., 2009). When it comes to group categorization, the clustering approach used in the present work to design groups based on contrasting dietary patterns was already used by other authors (Elisabet WirfäLt and Jeffery, 1997). Further studies with a larger sample size and more heterogeneous volunteer population should be done to highlight interindividual variations in terms of food preferences and choices and compare the respective weight of individual factors, habits, and cultural beliefs. In terms of methodology, it is also important to investigate the impact of potential confounding factors, such as dish familiarity or image physical properties. For example, Sokol-Hessner et al. (2012) showed in an fMRI study that the gaze contributes to valuating the fixated item more than the non-fixated item. Such results necessitate familiarizing beforehand the volunteers with the pictures (as we did in our pilot study) and pairing pictures with caution using the metadata provided in our database as in the Food-pics database (Blechert et al., 2014, 2019).

Compared to the SL condition, DL elicited dACC, anterior and posterior insula, thalamus, and occipital-fusiform complex activation. The dACC is known to be involved in internal conflict monitoring during choice task situation (Botvinick et al., 1999; Paus, 2001; Walton et al., 2003), matching our hypothesis and assuming that the DL condition elicited self-control dilemma between tasty and healthy foods. Insula has been identified as a key area in food reward anticipation (Pelchat et al., 2004; Beaver, 2006; Parigi et al., 2006). Co-activation of anterior and posterior insula in DL condition could be interpreted as taste anticipation and evaluation in regard to segregated roles of anterior and posterior insula. A higher awareness (Craig, 2002) toward taste anticipation processing (Rolls, 1997; Small, 2006) may be driven by the anterior insula to evaluate the different hedonic values of the two food pictures. A role of the posterior insula in caloric assessment is hypothesized because this area is known to be involved in energy detection (Beaver, 2006; Stoeckel et al., 2008) and in gastric distension perception (Tomasi et al., 2009). The FG was activated in both SL and DL situations, but the DL situation showed fusiform-occipital complex activation, whereas the SL condition showed fusiform activation alone. The FG has been implicated in focused visual attention (Vuilleumier and Driver, 2007) and plays a role in increasing salience of relevant behavior. In eating behavior context, it has been shown to be more activated when subjects are hungry (LaBar et al., 2001). With the occipital cortex, which is part of the visual associative areas involved in object recognition (Grill-Spector et al., 2001), the FG constitute the LOC, classically known as occipital FG under statistical parametric mapping (SPM). van der Laan et al. (2011) suggested that the LOC could be involved in more extensive visual processing and heightened attention required by food visual stimuli, notably because of its emotional salience. All this processing is hypothesized to be monitored by a top-down regulation assumed by the anterior cingulate (and amygdala) that projects to the LOC to control motivational state. The effectiveness of self-control dilemma could explain the higher monitoring by the LOC instead of the fusiform alone.

Compared to HE, the LE choices involved the HPC, FG, and supramarginal gyrus and seemed to require more complex brain processing than HE choices. When concomitantly activated with the HPC (and amygdala), the FG regulates goal-oriented behaviors by signaling relevant sensory cues according to motivational needs, that is, visceral states and homeostasis (LaBar et al., 2001; Malik et al., 2008). This synergy is made possible by hypothalamic projections to the amygdala and previous food experience memory retrieval insured by the HPC. Hippocampal memory retrieval and attentional focusing exerted by the FG could help the amygdala in positive or negative emotional attribution (Baxter and Murray, 2002) to food cues (e.g., negative valence of unhealthy energy-dense food), leading to an LE healthy food choice. Finally, the supramarginal gyrus is bordered by the lateral intraparietal area (LIP) known, in monkeys, to be involved in evidence accumulation during decision building and acts as an information store characterized by an increasing neuronal firing until a bound that elicits final decision in a two-choice task (Churchland et al., 2008).

## Conclusion and Perspectives

This work allowed us to implement a new food picture database with food items typical of institutional catering in France, suitable for future behavioral and neuroimaging experiments related to eating preferences, habits, and choices. Correspondences between real and estimated energy content of the food pictures and between energy content and individual liking were described in our volunteers, highlighting variations according to individual eating habits and notably to consumption frequency of “unhealthy” high-caloric palatable food items. Our findings confirm that the SL and DL conditions involved different neuronal networks, and that final HE or LE choice was linked to well-segregated cognitive processes. These results validated our experimental procedure and the implementation of our new food picture database in a cognitive task under fMRI, which is currently investigated in a homogeneous cohort study composed of 50 healthy normal-weight young women. In the future, we plan to increment our food picture database with season-specific fruits, vegetables, and dishes, as well as with diet-specific (e.g., vegetarian, etc.) and culture-specific dishes (e.g., foreign traditional dishes, etc.). Validation of the database with a larger and more diversified volunteer population is also expected to highlight preference variations according to age, gender, cultural, and alimentary taboos.

## Data Availability Statement

The datasets generated for this study are available on request to the corresponding author.

## Ethics Statement

This study was approved by the Ethics Committee Ouest V, Rennes (Comité de Protection des Personnes Ouest V), reference number: 11/43-832, study number: 2011-A01531-40. Prior to the experiment, subjects were given detailed information about the procedure and provided written informed consent.

## Author Contributions

DV-L secured the funding for this project. DV-L, RM, AC, PM, and NC designed the research. PM built the food picture database. YG built the screening questionnaire and recruited participants. PM and EB contributed to the fMRI task setup, and fMRI acquisition and analysis. J-CF performed the radiological reading. AC created the numeric form of the food consumption frequency questionnaire, and liking and caloric numeric scale questionnaires performed on TypeForm^®^. YG, PM, NC, and DV-L performed the research. YG and PM analyzed the data. YS produced the brain activations illustrations. YG, PM, and DV-L wrote the manuscript. All authors read and revised the manuscript.

## Conflict of Interest

The authors declare that the research was conducted in the absence of any commercial or financial relationships that could be construed as a potential conflict of interest.

## Abbreviations

AUDIT: alcohol use disorders identification test
BA: Brodmann area
BMI: body mass index
BOLD: blood oxygen level dependent
CRAFFT: car, relax, alone, forget, friends, trouble
dACC: dorsal anterior cingulate cortex
DL: different liking
FCFQ: food consumption frequency questionnaire
FG: fusiform gyrus
fMRI: functional magnetic resonance imaging
HAC: hierarchical ascendant classification
HE: high energy
HPC: hippocampus
LE: low energy
LOC: lateral occipital complex (also known as occipital fusiform gyrus)
PCA: principal component analysis
PTc: prudent-type consumers
SCOFF: sick, control, one stone, fat, food
SL: similar liking
WTc: western-type consumers.
